# Multimodal MR-imaging reveals large-scale structural and functional connectivity changes in profound early blindness

**DOI:** 10.1371/journal.pone.0173064

**Published:** 2017-03-22

**Authors:** Corinna M. Bauer, Gabriella V. Hirsch, Lauren Zajac, Bang-Bon Koo, Olivier Collignon, Lotfi B. Merabet

**Affiliations:** 1 Laboratory for Visual Neuroplasticity. Massachusetts Eye and Ear Infirmary, Harvard Medical School, Boston, MA, United States of America; 2 Center for Biomedical Imaging. Boston University School of Medicine, Boston, MA, United States of America; 3 Crossmodal Perception and Plasticity Laboratory. University of Trento, Trento, Italy; Chinese Academy of Sciences, CHINA

## Abstract

In the setting of profound ocular blindness, numerous lines of evidence demonstrate the existence of dramatic anatomical and functional changes within the brain. However, previous studies based on a variety of distinct measures have often provided inconsistent findings. To help reconcile this issue, we used a multimodal magnetic resonance (MR)-based imaging approach to provide complementary structural and functional information regarding this neuroplastic reorganization. This included gray matter structural morphometry, high angular resolution diffusion imaging (HARDI) of white matter connectivity and integrity, and resting state functional connectivity MRI (rsfcMRI) analysis. When comparing the brains of early blind individuals to sighted controls, we found evidence of co-occurring decreases in cortical volume and cortical thickness within visual processing areas of the occipital and temporal cortices respectively. Increases in cortical volume in the early blind were evident within regions of parietal cortex. Investigating white matter connections using HARDI revealed patterns of increased and decreased connectivity when comparing both groups. In the blind, increased white matter connectivity (indexed by increased fiber number) was predominantly left-lateralized, including between frontal and temporal areas implicated with language processing. Decreases in structural connectivity were evident involving frontal and somatosensory regions as well as between occipital and cingulate cortices. Differences in white matter integrity (as indexed by quantitative anisotropy, or QA) were also in general agreement with observed pattern changes in the number of white matter fibers. Analysis of resting state sequences showed evidence of both increased and decreased functional connectivity in the blind compared to sighted controls. Specifically, increased connectivity was evident between temporal and inferior frontal areas. Decreases in functional connectivity were observed between occipital and frontal and somatosensory-motor areas and between temporal (mainly fusiform and parahippocampus) and parietal, frontal, and other temporal areas. Correlations in white matter connectivity and functional connectivity observed between early blind and sighted controls showed an overall high degree of association. However, comparing the relative changes in white matter and functional connectivity between early blind and sighted controls did not show a significant correlation. In summary, these findings provide complimentary evidence, as well as highlight potential contradictions, regarding the nature of regional and large scale neuroplastic reorganization resulting from early onset blindness.

## Introduction

Ocular blindness has served as an important model in helping to understand the consequences of sensory deprivation on brain development. Extensive work in animal models has provided compelling anatomical and behavioral evidence regarding the dramatic neuroplastic changes that result from altering visual experience (e.g. [1–3]). In humans, there has been considerable interest in relating neuroplastic changes with compensatory behaviors observed in individuals living with profound blindness (see [4–6] for reviews). Indeed, there is mounting support that blind individuals (particularly, when blind from birth or very early in life) demonstrate comparable, and in some cases even superior, behavioral skills as compared to their sighted counterparts (e.g. [7–11]; for review see [12, 13]). Taken together, a contemporary view suggests that these compensatory behaviors may be intimately related to underlying changes in the overall structural and functional organization of the brain [14]. This reorganization implicates areas responsible for the processing of intact senses such as touch, hearing, and smell [15–17]. At the same time, there is also evidence of crossmodal reorganization within occipital cortex; that is to say, the area of the brain normally ascribed to processing visual information. Specifically, numerous neuroimaging studies have demonstrated that blind individuals show robust activation within occipital cortical areas while performing a variety of nonvisual tasks (e.g. Braille reading [18], sound localization [19–21], and odor perception [22]), as well as higher order cognitive tasks including language processing [23–25] and verbal memory recall [7, 26].

Despite accumulating evidence of these dramatic neuroplastic changes (and in particular implicating occipital cortical areas), a number of reports have suggested that the functional organization (i.e. domain specificity) of the occipital cortex may develop independently of visual experience (e.g. [27–31]). Importantly, it has been argued that this organization is maintained by an intrinsic pattern of anatomical and functional connectivity between occipital and other brain regions that process non-visual properties within corresponding cognitive domains (the “connectivity-constraint hypothesis”; see [31–33]). However, it remains unclear as to how an unaltered connectivity profile would in turn support compensatory behaviors in relation to crossmodal processing and reconcile the accumulating evidence of extensive functional reorganization described above. At this juncture, it would be reasonable to posit whether current views and interpretations are largely driven by focused analytic strategies (i.e. region of interest rather than a whole brain approaches) and inherent limitations related to data obtained from independently acquired imaging modalities.

At the regional level, structural morphometry studies of the occipital cortex in blind humans show evidence of decreased gray matter volume [14, 34–36] as well as concomitant increases in cortical thickness [34, 37–40]. Findings provided from diffusion based imaging studies (i.e. diffusion tensor imaging, or DTI) have consistently reported wide spread reductions in the structural integrity of geniculocalcarine structures and tracts such as the optic radiations [35, 36, 41–44], as well as a general trend of decreased overall connectivity throughout the brain [43, 45–47]. Analysis based on resting state functional connectivity MRI (rsfcMRI) has also been used to characterize large-scale functional network properties in the absence of task performance [48–50] but with mixed results. This includes reports of enhanced functional connectivity between occipital areas and other regions of the brain including parietal and frontal areas [23, 47, 51–54] while other studies have suggested patterns of overall decreases in connectivity between occipital areas and somatosensory cortex as well as temporal cortical areas implicated with auditory processing [23, 31, 53, 55, 56] (see also [57] for recent review). Furthermore, in the case of reported enhanced functional connectivity (e.g. between occipital and frontal regions), there appears to be a lack of evidence of concomitant increases in white matter connections that may putatively support crossmodal sensory and cognitive processing.

Certainly, these results continue to raise interesting questions regarding the nature and extent of neuroplastic changes within the context of profound blindness. However, uncertainty remains regarding the relationship between changes in brain structure and large-scale changes in anatomical and functional brain connectivity. This is particularly evident when one considers that previous imaging studies have usually focused on analyzing one parameter at a time, thus making reconciliation of observed results across multiple studies particularly challenging. Given these gaps in our understanding, it would be of value to capture multiple measures of morphometry, structural, and functional data throughout the brain as a means to better characterize the nature and extent of these changes resulting from visual sensory deprivation.

To our knowledge, no previous study has attempted to address this issue using a comprehensive multimodal imaging approach. Here, we investigated potential differences between early blind and sighted control individuals with respect to morphometry obtained from standard anatomical MRI, white matter connectivity and integrity obtained by diffusion MRI, as well as functional connectivity characterized by resting state fMRI. In so doing, we aimed to examine the interrelationship between these multiple imaging parameters, with particular attention to structural and functional connectivity.

## Materials and methods

### Study participants

A total of 28 subjects were recruited for the study and separated into two groups comprised of 12 early blind (6 females, mean age 33.58 years ± 7.51 S.D.) and 16 normally sighted controls (8 females, mean age 30.44 years ± 5.84 S.D.). Comparing demographic factors between both groups revealed no statistically significant differences in terms of age (p = 0.26) or gender (Chi-square = 0.86). For the purposes of this study, we defined “early blind” as documented residual vision no greater than light perception and/or hand motion acquired prior to the age of three (i.e. prior to the recall of visual memories and the development of high level language function; see [58, 59]). While the majority of participants had diagnoses that could be considered as a “congenital” cause, we relied on documented clinical evidence of profound blindness based on a structured and functional assessment. The etiologies of blindness were varied and included retinal dystrophies as well as ocular malformations. However, no single diagnosis was represented in more than three subjects. All blind participants were highly independent travelers, employed, college educated, and experienced Braille readers. They were predominately right handed (based on self-report), but most used two hands for the purposes of reading Braille text (see Table 1: Subject Demographics for complete details regarding the demographics of the blind participants). Sighted controls had normal, or corrected-to-normal, visual acuity. Apart from blindness, the participants had no documented history of neurological abnormalities. Written informed consent was obtained from all subjects prior to participation and all experimental procedures were approved by the Institutional Review Board at the Massachusetts Eye and Ear Infirmary, Boston, MA, USA.

**Table 1:** Subject Demographics.

subject	age	gender	Braille reader	blindness onset	level of residual vision	diagnosis
1	33	M	Yes	Birth	LP	congenital retinitis pigmentosa
2	29	M	Yes	Birth	NLP	congenital anophthalmia
3	38	M	Yes	Birth	LP	Leber’s congenital amaurosis
4	44	M	Yes	Birth	NLP	congenital optic nerve atrophy
5	41	M	Yes	Birth	NLP	Leber’s congenital amaurosis
6	37	M	Yes	3 y.o.	LP	juvenile macular degeneration/glaucoma
7	25	F	Yes	Birth	LP	Leber’s congenital amaurosis
8	28	F	Yes	3 y.o.	NLP	unknown ocular cause
9	29	F	Yes	Birth	LP	retinopathy of prematurity
10	30	F	Yes	Birth	LP	retinopathy of prematurity
11	46	F	Yes	Birth	LP	ocular infection
12	23	F	Yes	Birth	LP	retinopathy of prematurity

### MRI acquisition

All imaging data were acquired on a 3T Philips Achieva System (Best, the Netherlands) with an 8-channel phased array coil. Subjects were instructed to lie still and foam padding was used to minimize head motion. Two structural T_1_-weighted scans were acquired using a turbo spin echo sequence (TE = 3.1 ms, TR = 6.8 ms, flip angle = 9°, voxel size 0.98 x 0.98 x 1.20 mm). Diffusion based imaging was carried out using high angular resolution diffusion imaging (HARDI). Images were acquired with a single-shot EPI sequence (TE = 73 ms, TR = 17844 ms, flip angle 90°, 64 directions, EPI factor = 59, B0 = 0 s/mm^2^, Bmax = 3000 s/mm^2^, voxel size 1.75 x 1.75 x 2.00 mm, enhanced gradients at 66 mT/m, and a slew rate of 100 T/m/ms). Resting state functional connectivity (rsfcMRI) was acquired with a 7 min single-shot EPI sequence (TE = 30 ms, TR = 3000 ms, flip angle = 80°, voxel size 2.75 x 2.75 x 3.00 mm, prospective motion correction). For the resting state sequence, subjects were blindfolded and instructed to lie still and let their minds “wander” while remaining awake. Finally, a field map was acquired to correct for EPI related field inhomogeneities using a fast field echo sequence (TE1 = 2.3 ms, TE2 = 4.6 ms, TR = 20 ms, flip angle = 10°, voxel size 1.02 x 1.02 x 3.00 mm).

### Data processing

**Fig 1:**
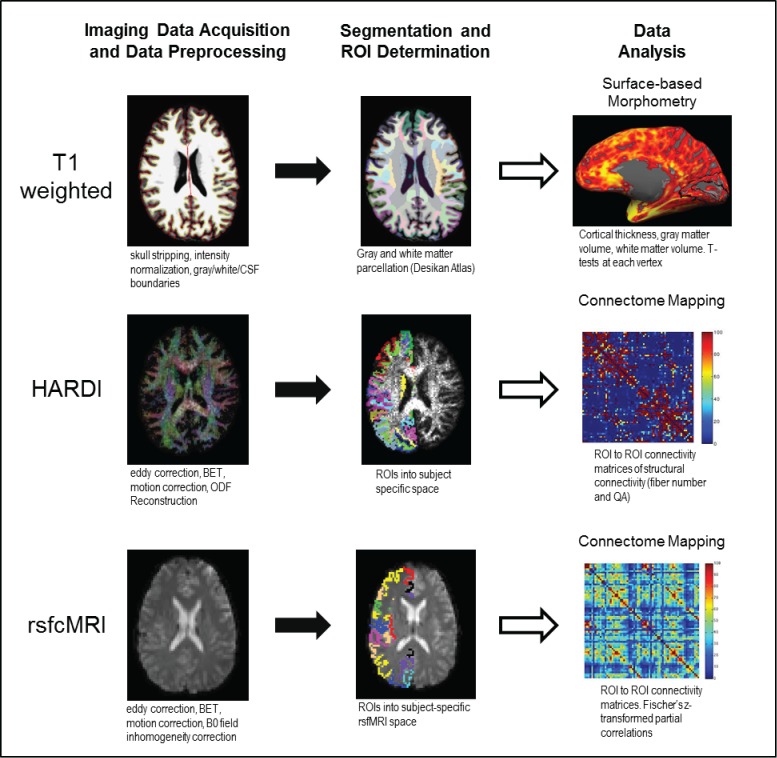
Summary of processing stream for T_1_ weighted morphometry, HARDI white matter tractography, and rsfcMRI functional connectivity analyses. Following standard preprocessing, gray and white matter were divided into anatomically-derived parcellations (Desikan atlas). Surface based morphometry (SBM) was used to analyze cortical thickness and volume as well as the volume of subcortical gray matter and white matter regions. Following preprocessing of HARDI data, the orientation distribution function (ODF) for each voxel was calculated. The gray and white matter segmentations from the Desikan atlas were transformed into HARDI space and used as seeds for ROI to ROI tractography. Resting state connectivity data were preprocessed and ROIs from the Desikan atlas were transformed into rsfcMRI space for each subject and used as seeds for ROI to ROI analysis. For both HARDI and rsfcMRI data, connectivity matrices for each subject were created from which group average matrices were made.

Data processing procedures and analysis workflow for structural morphometry, HARDI-based tractography, and rsfcfMRI analyses are outlined in Fig 1. Further details are provided below.

#### Image preprocessing and surface-based morphometry

Anatomical T_1_-weighted scans were processed using FreeSurfer 5.3.0 (https://surfer/nMR/mgh/harvard.edu). Details regarding this procedure have been described elsewhere [60]. Briefly, two structural T_1_-weighted scans were co-registered, intensity normalized, skull stripped, and the gray matter and white matter surfaces were defined based on intensity gradients. The accuracy of brain extraction and gray and white matter surfaces were confirmed by visual inspection and manual edits were applied when necessary. The cortex was parcellated into 68 discrete regions (34 per hemisphere) according to the Desikan atlas [61]. For surface-based analyses, gray matter cortical thickness and volume were calculated for each vertex. White matter segmentations for HARDI tractography were estimated 2 mm into the white matter from the gray/white matter boundary for each of the overlying cortical gray matter parcellations. Subcortical gray matter structures were also segmented [62, 63]. Volume was calculated for subcortical and white matter regions as part of the standard FreeSurfer processing pipeline. As done in previous studies (e.g. [64–66]), estimated total intracranial volume was also calculated for each subject and mean-centered (i.e. with respect to the mean of all subjects).

#### HARDI preprocessing and structural connectivity matrix construction

HARDI data was skull-stripped and corrected for eddy currents using the brain extraction tool (BET) and Eddy-correct from FSL 5.0.8 (FMRIB Software Library, http://fsl.fMRib.ox.ac.uk/fsl). The orientation distribution function (ODF) was reconstructed in DSI-Studio (http://dsi-studio.labsolver.org) using generalized q-sampling imaging (GQI) [67] with a diffusion sampling length ratio of 1.25 and ODF sharpening via decomposition [68]. A decomposition fraction of 0.04 and maximum fiber population of 8 were used. Three fibers per voxel were resolved with an 8-fold ODF tessellation. HARDI data was co-registered to the corresponding T_1_-weighted structural image for each subject using boundary-based registration (BBR) in FreeSurfer [69]. Registration accuracy was verified for each subject by visual inspection and manual corrections were performed where necessary. Each of the 68 cortical and 68 white matter parcellations [61] for the T_1_-weighted image were reverse transformed into subject-specific HARDI space, creating the seed (start) and target (end) point regions of interests (ROIs) for tractography analysis. An in-house program utilizing the tractography function from DSI-Studio generated streamlines to produce a whole brain connectivity matrix symmetrized between each pair of ipsi- and contra-lateral ROI pairs. A 68 x 68 connectivity matrix was generated using a termination angle of 45°, subject-specific quantitative anisotropy (QA) threshold (range 0.025 to 0.15, mean 0.054), smoothing of 0.5, step size 0.5 mm, minimum length = 5 mm, maximum length = 300 mm, random fiber direction, Gaussian radial interpolation, and 100,000 seeds. To capture the maximum possible number of streamlines associated with each cortical region, the start ROI was placed in the white matter. To ensure that the target cortical region was reached, the end ROI was placed in gray matter. Any circular fibers were automatically removed as part of the algorithm, as were tracts that extended beyond the target and seed ROIs. Two areas were considered to be connected if one or more fibers were present between them [70]. Individual subject matrices were generated for the number of streamlines/fibers between ROIs as well as average QA for each connection. Similar to the fractional anisotropy (FA) measure obtained in DTI, QA is an analogous metric obtained with HARDI and is an indicator of overall white matter structural integrity [67]. Average QA was calculated by sampling the HARDI maps at each step of the selected streamline.

#### rsfcMRI preprocessing and connectivity matrix generation

Resting state data was pre-processed with FEAT v. 6.00 (FMRI Expert Analysis Tool) from FSL. Preprocessing steps included removal of non-brain tissue using BET, B0 unwarping in the -x direction, and motion correction using MCFLIRT. To further control for the effects of motion, 6 motion covariates and their temporal derivatives were regressed out of the resting state signal, discarding any motion outlier data. Prior to applying motion correction, individual head movement was quantified and visually assessed for each subject. No subjects exceeded 0.27 mm absolute or relative motion (frame to frame measure of displacement), which was well below the acquired voxel size of 3 mm. Subsequent analysis confirmed that there were no statistically significant differences in head motion between the two groups (mean sighted controls = 0.10 mm absolute, 0.08 mm relative and mean early blind = 0.15 mm absolute, 0.11 mm relative; p>0.05). rsfcMRI data were co-registered using BBR [69] to the anatomical T_1_-weighted scan. Cortical parcellations [61] were reverse transformed into subject-specific rsfcMRI space. An in-house MATLAB program was used to calculate temporal partial correlations between ROIs. A high pass filter of 0.01 Hz and a low pass filter of 0.1 Hz were applied. Data were also de-trended and Fischer’s z-transformed to ensure normality. Similar to HARDI data, a 68 x 68 symmetrized connectivity matrix was generated for each subject whereby the z-transformed correlation coefficient between each parcellated region (node) was stored at a unique position in the matrix and representing the functional connectivity strength between nodes.

### Statistical analysis

Statistical analyses on surface based morphometry was carried out at two levels. First, an exploratory analysis regarding potential group differences in cortical thickness and volume were evaluated using a significance threshold of p<0.005 (two-tailed, uncorrected) as was done in previous studies (e.g. [14, 40]). Volume measures were corrected for mean-centered intra-cranial volume to account for the potential effect of head size (e.g. [64–66]). A second level of analysis was performed by fitting a general linear model (GLM) in FreeSurfer. A series of 10,000 Monte Carlo simulations were then performed on the resulting data using a vertex-size threshold of p<0.005 and a cluster-wise threshold of p<0.05. For both levels of group comparisons, individual surfaces were registered to standard space and spatially smoothed using a Gaussian kernel of 5 mm full width half maximum (FWHM). The volume of each subcortical or white matter region was corrected using residuals of intra-cranial volume (e.g [71–73]). Differences between groups in subcortical and white matter volume were analyzed using SAS (University Edition).

To assess differences in connectivity (HARDI and rsfcMRI) between individuals in the sighted and blind groups, a two-sample t-test was performed for each connection between pairs of ROIs. This resulted in a total of 2278 (i.e. 68 X 67/2 = 2278) connections. A False Discovery Rate (FDR) analysis was performed at a rate of q = 0.05 to correct for multiple comparisons within the structural and functional connectivity matrices [74] as previously done in past studies [75, 76].

As a final analysis, a Pearson’s r-coefficient was used to determine the degree of association between the acquired measures of structural (number of fibers obtained by HARDI) and functional (partial correlations obtained by rsfcMRI) connectivity. Prior to correlation analyses, HARDI matrices were z-transformed to ensure normality. Correlations between early blind and sighted control subjects for both white matter fiber number and resting state connectivity were examined separately. Finally, to explore if alterations in the profile of white matter connectivity were related to alterations in the profile of resting state functional connectivity, correlations were determined between changes in structural connectivity (i.e. early blind and sighted controls) with changes in functional connectivity (i.e. early blind and sighted controls). For this correlation analysis, the difference between groups was calculated by subtracting the mean sighted control connectivity data from the mean ocular blind connectivity data (separately for HARDI and for rsfcMRI data). These difference matrices were z-transformed prior to the final correlation analysis.

### Construction of circular connectograms

The term connectome has been used to refer to the development of comprehensive maps characterizing neural connections within the brain [77]. In this study, we employed the circular connectogram to depict large-scale structural and functional connectivity patterns in early blind and sighted controls as well as differences between these two groups. The format is based on the freely available Circos software ([78]; http://www.circos.ca/) and processing pipelines adapted for neuroimaging data have been previously explained in detail [79]. Briefly, the circular connectogram design allows for the depiction of regional structural and inter-regional connectivity data within the same two-dimensional graphical representation. The circular layout facilitates the display of relationships between pairs of positions by using color coded links (in the case of HARDI and rsfcMRI connectivity data) and heat maps (in the case of morphometry data) presented along a circular array of radially aligned cortical parcellations (the “connectogram” [79];).

## Results

### Surface-based morphometry

An initial exploratory analysis (uncorrected, p<0.005) revealed widespread differences (i.e. trends of both increases and decreases) in cortical volume throughout both hemispheres when comparing early blind to sighted controls. Specifically, clusters showing increases in cortical volume were observed bilaterally in the temporal, frontal, cingulate, and motor (i.e. precentral gyrus) cortices, as well as the right parietal cortex. Decreases in cortical volume in early blind were more widespread and clusters showing decreases in volume were observed bilaterally in the occipital, temporal, parietal, and frontal, as well as the left cingulate and sensorimotor (i.e. pre-and post-central gyri) cortices (p<0.005 uncorrected). In the subsequent analysis, only two of these clusters survived correction for multiple comparisons. Specifically, a significant decrease in volume of the left pericalcarine cortex (p = 0.0066) and a significant increase in volume of the right inferior parietal cortex (p = 0.0032) was evident when comparing early blind to sighted controls (Table 2: Surface Based Morphometry Analysis (corrected); see also supplementary materials 1).

**Table 2:** Surface Based Morphometry Analysis (corrected).

Cortical Volume								
	Region Label	Cluster size (mm^2^)	MNI Coordinates			adjusted p value	Lower C.I.	Upper C.I.
			x	y	z			
**early blind < sighted controls**								
**Left**	pericalcarine	216.82	-8.9	-91	7.1	0.0066	0.0052	0.0080
**early blind > sighted controls**								
**Right**	inferior parietal	241.25	47	-56.9	14.2	0.0032	0.0022	0.0042
**Cortical Thickness**								
	Region Label	Cluster size (mm^2^)	MNI Coordinates			adjusted p value	Lower C.I.	Upper C.I.
			x	y	z			
**early blind < sighted controls**								
**Left**	fusiform	143.79	-39.4	-42.1	-22.7	0.0288	0.0258	0.0317

Trends for widespread increases and decreases in cortical thickness were also observed following the initial exploratory analysis. Specifically, clusters showing increased cortical thickness in the ocular blind compared to sighted controls was observed bilaterally in the occipital, temporal, and parietal lobes. Clusters showing decreased cortical thickness in the ocular blind were observed bilaterally in the occipital, temporal, frontal, and parietal lobes (p<0.005 uncorrected). However, the only cluster to survive correction for multiple comparisons was a significant decrease observed in the left fusiform gyrus (Table 2: Surface Based Morphometry Analysis (corrected); see also S1 Fig).

Exploratory analysis (uncorrected, p<0.005) of changes in white matter volume revealed that early blind subjects showed decreases compared to sighted controls within occipital regions including bilateral pericalcarine, cuneus, and lingual cortices, as well as the right lateral occipital region. There was no significant trend for relative increases in white matter volume in blind subjects compared to controls.

Finally, comparing subcortical structures (specifically, the hippocampus, amygdala, caudate, and putamen) did not reveal statistically significant differences between early blind and sighted controls (all p>0.005). Complete results from the morphometric analyses (uncorrected) are reported in S1 Table.

### HARDI white matter structural connectivity

**Fig 2:**
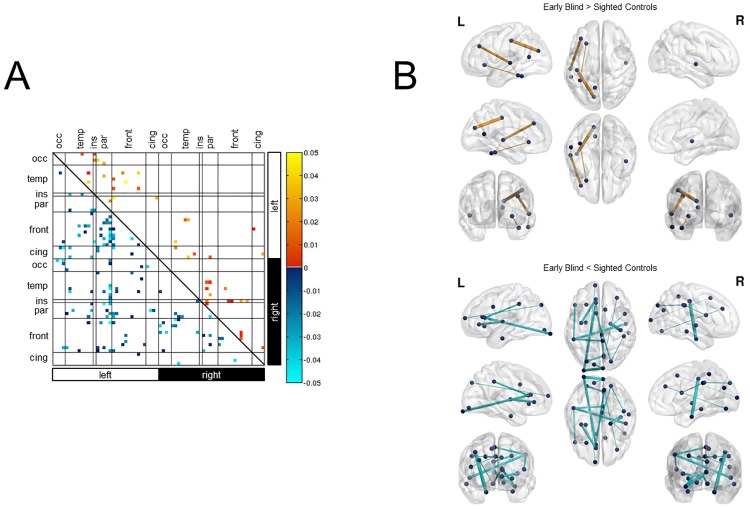
White matter structural connectivity revealed by HARDI. A) Exploratory analysis (uncorrected; p<0.05) of ROI-pairs revealed trends for increased as well as decreased white matter connectivity (as indexed by fiber number) in early blind compared to sighted control individuals. These included increases between occipital and frontal cortices, motor (i.e. precentral gyrus), and somatosensory regions (i.e. postcentral gyrus). B) Ball and stick representation of the increases and decreases in white matter fiber number between early ocular blind and sighted controls following FDR correction. Differences in connectivity strength are represented by line thickness, whereby thicker lines represent larger differences between the two groups (based on p-value). Increases are represented by orange lines, whereas decreases are represented by cyan lines. Dark blue spheres represent the nodes (i.e. ROIs) associated with the start and/or end points of the connections. A total of 5 connections showed an increase in fiber number after FDR correction (upper panel). These were mainly in the left hemisphere and included fronto-temporal, as well as parieto-precentral connections. A total of 18 connections survived FDR multiple comparisons correction for the decreased fiber number in early ocular blind subjects (lower panel). These were evenly distributed between the hemispheres and included significant decreases between occipital and frontal cortices, as well as the entorhinal and precentral regions. Abbreviations: L = left, R = right, cing = cingulate, front = frontal cortex, par = parietal cortex, ins = insula, temp = temporal cortex, occ = occipital cortex.

Exploratory analysis of ROI-pairs revealed a number of connections that differed significantly in early blind subjects compared to sighted controls (p<0.05). Specifically, increased connectivity (indexed by fiber number) in early blind compared to sighted was observed for bilateral intra-hemispheric and inter-hemispheric connections involving temporal, parietal, and frontal lobes, as well as the left occipital and sensorimotor cortices (Fig 2A). Five of these connections survived FDR correction for multiple comparisons. These were mainly left-lateralized and evident between temporal and frontal regions, as well as between primary motor and the precuneus (Fig 2B). Exploratory analysis also revealed a number of connections showing decreased fiber number in the ocular blind group compared to controls throughout the entire cortex. These were inter- and bilateral intra-hemispheric connections involving the occipital, temporal, parietal, frontal, cingulate, and sensori-motor cortices (Fig 2A). Decreased connectivity was observed between 18 connections after FDR correction for multiple comparisons. These were equally distributed across both hemispheres. Within the left hemisphere, and the majority of decreases involved the frontal lobe (e.g. frontal to primary sensory, fronto-frontal, fronto-insular, and fronto-parietal cortices). An additional connection demonstrating decreased structural connectivity in early blind was noticed between the occipital lobe and cingulate cortex. Additional decreases included right intrahemsipheric connections also implicated the frontal lobes (e.g. occipito-frontal and frontal-cingulate) as well as the sensori-motor cortices. Concerning interhemispheric connections, decreases in occipito-occipital connections were most significant (Fig 2B). Connections surviving FDR correction are outlined in Table 3: HARDI White Matter Connectivity (corrected).

**Table 3:** HARDI White Matter Connectivity (corrected).

Fiber Number (HARDI)								
**early blind < sighted controls**								
Left intrahemispheric			Interhemispheric			Right intrahemispheric		
ROI 1	ROI 2	adjusted p value	ROI 1	ROI 2	adjusted p value	ROI 1	ROI 2	adjusted p value
**occipital**			**occipital**			**occipital**		
lh lateral occipital	lh rostral anterior cingulate	0.0096	lh lateral occipital	rh cuneus	0.0084	rh lingual	rh rostral middle frontal	0.0221
			rh lingual	lh rostral anterior cingulate	0.0151	rh cuneus	rh superior frontal	0.0237
**parietal**			lh pericalcarine	rh cuneus	0.0186			
lh superior parietal	lh frontal pole	0.0217				**frontal**		
			**temporal**			rh pars opercularis	rh posterior cingulate	0.0212
**frontal**			lh entorhinal	rh middle temporal	0.0240			
lh medial orbitofrontal	lh insula	0.0105				**sensori-motor**		
lh pars triangularis	lh lateral orbitofrontal	0.0234	**parietal**			rh precentral	rh entorhinal	0.0042
			lh superior parietal	rh precuneus	0.0138	rh postcentral	rh superior parietal	0.0204
**sensori-motor**			rh superior parietal	lh caudal anterior cingulate	0.0172			
lh postcentral	lh pars triangualris	0.0145						
			**sensori-motor**					
			lh postcentral	rh superior temporal	0.0107			
			lh postcentral	rh paracentral	0.0142			
**early blind > sighted controls**								
Left intrahemispheric			Interhemispheric			Right intrahemispheric		
ROI 1	ROI 2	adjusted p value	ROI 1	ROI 2	adjusted p value	ROI 1	ROI 2	adjusted p value
**temporal**			**temporal**					
lh superior temporal	lh rostral middle frontal	0.0112	rh superior temporal	lh retrosplenial	0.0205			
lh fusiform	lh lateral orbitofrontal	0.0185						
lh superior temporal	lh inferior temporal	0.0198						
**sensori-motor**								
lh precentral	lh precuneus	0.0082						

An analysis of QA was also carried out as an index of white matter integrity. Exploratory analysis of ROI-pairs revealed a number of connections that differed for QA in early blind subjects compared to sighted controls (p<0.05). Specifically, increased QA was observed in early blind compared to sighted for bilateral intra-hemispheric connections involving the occipital, temporal, parietal, frontal, and cingulate cortices. Additional inter-hemispheric increases in QA were also observed, however these were less numerous than intra-hemispheric increases. Increased QA was observed in five connections following FDR correction for multiple comparisons. Increases in QA in the early blind compared to sighted were mainly left lateralized involving fronto-temporal and temporal-temporal white matter connections. Exploratory analysis also revealed a number of connections showing decreases in QA in early blind compared to sighted subjects (p<0.05). These were equally distributed between inter-and intra-hemispheric connections and involved occipital, temporal, parietal, frontal, and cingulate cortices. Decreased QA was observed in 14 connections after FDR correction for multiple comparisons. Decreases in QA included the interhemispheric connections between the occipital lobes through the splenium were among those that survived FDR correction. Further decreases surviving FDR correction included the left occipito-cingulate, left entorhinal to insula, as well as right lingual to parahippocampal gyrus, and right entorhinal to precentral gyrus (see S2 Fig and S2 Table for a complete description of connections surviving FDR correction relating to QA).

### Resting state functional connectivity

**Fig 3:**
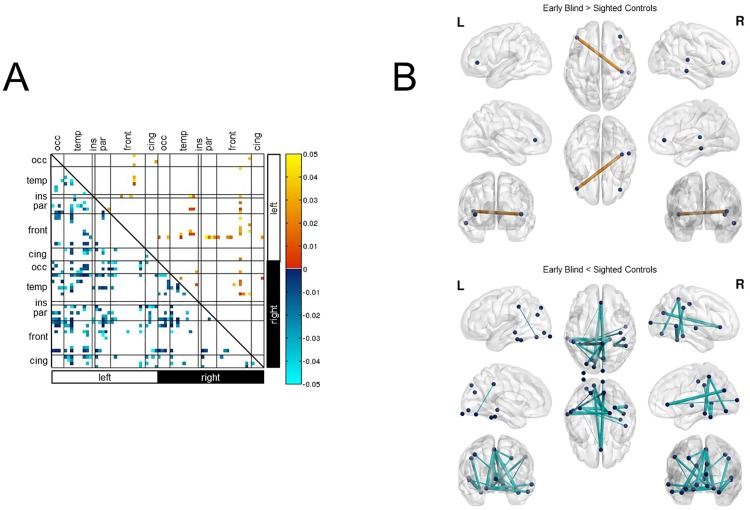
Functional connectivity revealed by rsfcMRI. A) Exploratory analysis (uncorrected; p<0.05) of ROI-pairs revealed widespread differences in functional connectivity between early blind and sighted control individuals. While patterns of increases and decreases in functional connectivity were evident, the overall trend was for a decrease in resting state temporal correlations in early blind compared to sighted controls. This included between occipital-motor regions, occipital somatosensory connections, as well as decreases in occipito-frontal and occipito-memory-related structures. Increases in functional connectivity were evident bilaterally between occipital regions and the pars orbitalis in the inferior frontal lobe. Bilateral increases involving the pars orbitalis were also observed to portions of the temporal lobe involved with auditory processing (namely, the transverse temporal, banks of the superior temporal sulcus, superior temporal, and middle temporal cortices). B) Ball and stick representation of increases and decreases in resting state connectivity between early ocular blind and sighted controls following FDR correction. Differences in connectivity strength are represented by line thickness, whereby thicker lines represent larger differences between the two groups (based on p-value). Increases are represented by orange lines, whereas decreases are represented by cyan lines. Dark blue spheres represent the nodes (i.e. ROIs) associated with the start and/or end points of the connections. A total of two connections showed an increase in functional connectivity after FDR correction (upper panel). These were between right transverse temporal and left pars orbitalis, as well as the right middle temporal and right pars orbitalis. A total of 22 connections showing a decrease in functional connectivity survived correction (lower panel). These were mainly inter-hemispheric and occipital connections were centered around the fusiform gyrus, orbitofrontal regions, and sensorimotor cortices. Additional decreases within both left and right hemispheres were seen between occipital and primary sensori-motor cortices. Abbreviations: L = left, R = right, cing = cingulate, front = frontal cortex, par = parietal cortex, ins = insula, temp = temporal cortex, occ = occipital cortex.

Similar to the results obtained regarding white matter connectivity, an exploratory analysis of ROI-pairs of the rsfcMRI data also revealed a mixture of widespread increases and decreases of functional connectivity between the two groups of interest (p<0.05). Notably, increases in temporal correlations in early blind compared to sighted involved bilateral intra-hemispheric occipital, temporal, and frontal connections, as well as inter-hemispheric connections involving the occipital, temporal, frontal, and sensori-motor cortices (Fig 3A). Of the observed increases in functional connectivity, only two survived FDR correction for multiple comparisons. Specifically, these were temporal correlations between the right pars orbitalis and the right middle temporal lobe, and between the left pars orbitalis and the right transverse temporal gyrus (i.e. Heschl’s gyrus) (Fig 3B).

In the exploratory analysis, there was an overall greater trend for decreases rather than increases in temporal correlations between regions when comparing early blind to sighted. In particular, bilateral intra-hemispheric and inter-hemispheric connections involving the occipital, temporal, frontal, parietal, cingulate, and sensorimotor cortices (Fig 3A). Of the observed decreases in functional connectivity, a total of 22 survived FDR correction for multiple comparisons. Specifically, these involved bilateral decreases in connections between the lingual gyrus and primary somatosensory (i.e. post/paracentral gyri) cortex. Further decreases were observed between the right primary somatosensory cortex (i.e. post/paracentral gyri) and areas of the temporal lobe involved with spatial navigation and face recognition, namely the parahippocampal and fusiform gyri. Other notable inter-hemispheric decreases in functional connectivity were observed between occipital and sensorimotor regions, occipital and frontal regions, as well as between temporal (primarily fusiform and parahippocampal gyri) and sensorimotor areas. A listing of all significant connections following FDR correction can be found in Table 4: rsfcMRI Functional Connectivity (corrected).

**Table 4:** rsfcMRI Functional Connectivity (corrected).

rsfcMRI											
**early blind < sighted controls**											
Left intrahemispheric					Interhemispheric				Right intrahemispheric		
ROI 1		ROI 2		adjusted p value	ROI 1	ROI 2		adjusted p value	ROI 1	ROI 2	adjusted p value
**occipital**					**occipital**				**occipital**		
lh lingual		lh postcentral		0.0020	lh pericalcarine	rh paracentral		0.0006	rh lingual	rh paracentral	0.0010
					rh lingual	lh postcentral		0.0009	rh cuneus	rh medial orbitofrontal	0.0021
					lh pericalcarine	rh medial orbitofrontal		0.0014			
					lh lingual	rh paracentral		0.0020	**temporal**		
					lh lingual	rh medial orbitofrontal		0.0025	rh bank sts	rh fusiform	0.0000
					lh lateral occipital	rh fusiform		0.0025			
									**parietal**		
					**temporal**				rh precuneus	rh medial orbitofrontal	0.0002
					rh parahippocampal	lh inferior temporal		0.0008			
					rh parahippocampal	lh middle temporal		0.0009	**sensori-motor**		
					rh parahippocampal	lh superior parietal		0.0017	rh paracentral	rh parahippocampal	0.0003
					lh fusiform	rh superior temporal		0.0019	rh postcentral	rh fusiform	0.0008
					lh fusiform	rh medial orbitofrontal		0.0024			
					**parietal**						
					lh precuneus	rh medial orbitofrontal		0.0009			
					**sensori-motor**						
					rh paracentral	lh fusiform		0.0001			
					rh paracentral	lh superior parietal		0.0013			
					rh precentral	lh fusiform		0.0016			
**early blind > sighted controls**											
Left intrahemispheric					Interhemispheric				Right intrahemispheric		
ROI 1	ROI 2		adjusted p value		ROI 1		ROI 2	adjusted p value	ROI 1	ROI 2	adjusted p value
					**temporal**				**temporal**		
					rh transverse temporal		lh pars orbitalis	0.0012	rh middle temporal	rh pars orbitalis	0.0016

### Correlation between white matter and functional connectivity

**Fig 4:**
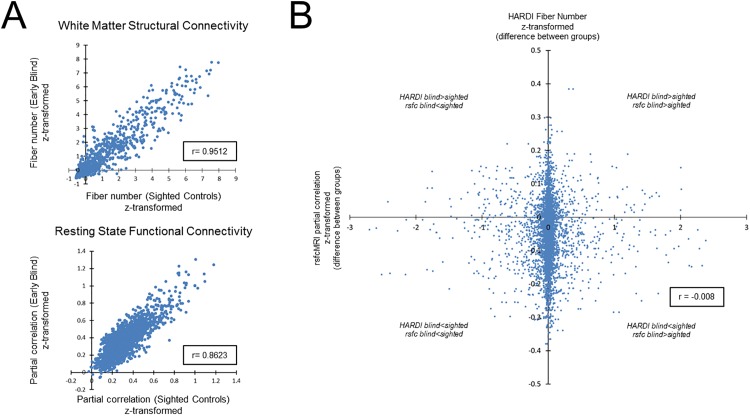
Correlation between white matter structural (obtained by HARDI) and resting state (obtained by rsfcMRI) functional connectivities. A) Scatter plot depicting the degree of association characterizing white matter structural (upper panel) and resting state functional (lower panel) connectivity between early blind and sighted controls. The measure of association (Pearson’s coefficient) was found to be highly significant (p<0.0001) for both white matter structural connectivity and resting state functional connectivity (r = 0.9512 and r = 0.8623, respectively). B) Scatter plot depicting correlations on all t-statistics of early blind compared to sighted controls for both white matter structural connectivity (HARDI fiber number) and functional connectivity (resting state partial correlations). Associating relative changes in white matter and functional connectivity between early blind and sighted controls were not statistically significant (r = -0.008, p>0.05).

As a final analysis, the degree of association characterizing structural connectivity (i.e. white mater fiber number obtained by HARDI) and functional connectivity (i.e. partial correlations obtained by rsfcMRI) between early blind and sighted controls was independently determined. Regarding white matter structural connectivity, the measure of association was found to be very high (r = 0.9512; p<0.0001) (Fig 4A upper panel). Similarly, the measure of association for resting state functional connectivity was also found to be very high (r = 0.8623; p<0.0001) (Fig 4A lower panel). Secondly, the degree of association between measured changes in structural connectivity and changes in functional connectivity between early blind and sighted controls was also determined. In this latter analysis, correlations on differences (i.e. early blind minus sighted controls) between early blind compared to sighted controls for both structural connectivity and functional connectivity were not statistically significant (r = -0.008, p = 0.5843) (Fig 4B).

### Circular connectogram visualization

**Fig 5:**
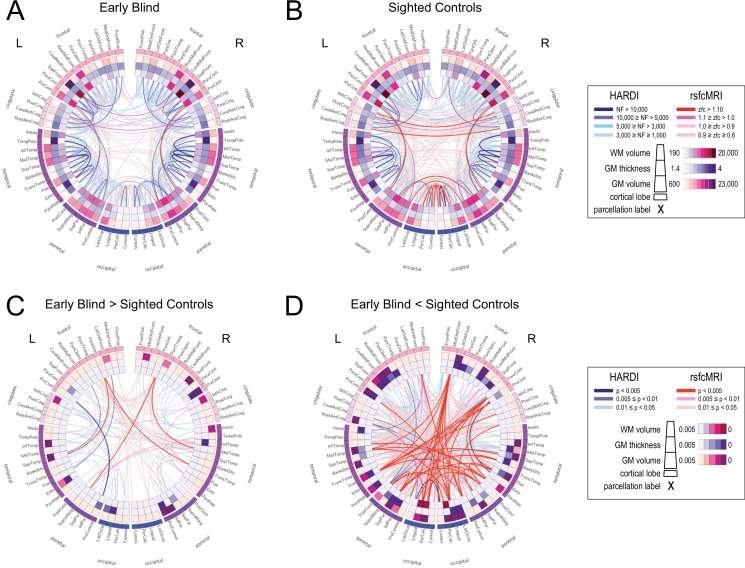
Circular connectograms illustrating regional differences in morphometry as well as white matter and functional connectivity networks in early blind (A) and sighted controls (B). The outermost ring corresponds to the various parcellated brains regions arranged by lobe and from anterior to posterior. The inner three rings (outermost to innermost) correspond to measures of gray matter volume, cortical thickness, and white matter volume (relative magnitudes shown in inset). The line segments in the center of the connectogram correspond to different levels in connectivity strength between parcellated brain regions. Blue lines represent streamlines between regions computed using HARDI white matter tractography. Red lines correspond to functional connections computed through performing partial correlations based on rsfcMRI data. Group average measures (uncorrected data) reveal trends across all imaging parameters in regional morphometry and connectivity profiles for early blind (A) and sighted controls (B) (absolute magnitudes shown in inset). Group differences between early blind and sighted controls are illustrated by relative increases (C) and decreases (D) in the blind compared to sighted controls (relative magnitudes shown in inset). See text for further details regarding connectome construction and description of morphometry and connectivity analyses.

To help better visualize the inter relationships between all the data modalities acquired, the results of the exploratory morphometry analysis (cortical thickness and volume, and white matter volume; all uncorrected), white matter connectivity (uncorrected), and functional connectivity (uncorrected) were presented simultaneously using a circular connectogram (individual group connectivity connectograms for early blind and sighted controls are shown in Fig 5A and 5B respectively).

In the early blind, visual inspection of the average group map revealed a trend for increased parietal-frontal white matter connections within the left hemisphere (Fig 5A). In contrast, sighted controls showed a trend for greater interhemispheric functional connectivity, particularly between occipital-occipital, cingulate-cingulate, and parietal-parietal cortices (Fig 5B).

Group differences in morphometry and connectivity were more evident using differential maps illustrating relative increases (Fig 5C) and relative decreases (Fig 5D) in early blind as compared to sighted controls. Specifically, increased number in white matter connections in the early blind (blue lines) were evident between occipital regions and parietal and temporal (predominately intrahemispheric with greater white matter connectivity evident within the left hemisphere). The greatest increases in white matter connectivity were between left parietal (precuneus) and precentral cortices as well as left temporal (fusiform and superior temporal) and frontal cortices (lateral orbital frontal and rostral middle frontal respectively). Increases in functional connectivity (intra- and interhemispheric) were also evident between occipital and other cortical areas (i.e. cingulate, frontal, and temporal cortices; indicated by red lines). The greatest increases (dark red lines) in functional connectivity were observed between bilateral pars orbitalis of the inferior frontal gyrus and the temporal and parietal lobes (specifically left superior temporal, right middle and transverse temporal, and the left supramarginal gyrus). The observed increases in cortical thickness in left lateral occipital cortex as well as additional increases in cortical thickness and volume are shown within the inner rings. In contrast, trends for overall decreases in white matter and functional connectivity in the early blind appeared to be largely inter-hemispheric. The most significant decreases in structural connectivity in early blind compared to sighted controls were observed between interhemispheric occipital regions (dark blue lines), as well as between the right precentral (i.e. motor) and entorhinal cortices. Regions showing multiple (i.e. ≥ four) significant changes in functional connectivity (dark red lines) included the right medial orbitofrontal cortex, paracentral lobe, and parahippocampus, as well as the left superior parietal and fusiform cortices, and the bilateral lingual gyrus. Finally, corresponding differences in gray and white matter morphometry between early blind and sighted controls can also be easily visualized confirming an overall trend for decreases in morphometry measures rather than increases (inner rings).

## Discussion

### Overview of results

In this study, we employed a multimodal MR-based imaging approach to provide multiple lines of evidence consistent with extensive morphological, structural, and functional reorganization within the brain and in the setting of early onset and profound ocular blindness. These observed changes were widely distributed, implicating areas responsible for the processing of intact sensory modalities (such as touch and hearing), occipital cortical regions (normally implicated in the processing of visual information), and regions involved in higher order cognitive functions (such as language, memory, and executive functions).

In summary, outcomes of morphometry in early blind compared to sighted controls revealed co-occurring decreases in cortical volume and cortical thickness within visual processing areas of the occipital and temporal cortices respectively. Increases in cortical volume in the early blind were evident within regions of parietal cortex (corrected results). White matter connectivity in the blind was increased predominantly within the left hemisphere, including between frontal and temporal areas implicated with language processing. Decreases in structural connectivity were evident involving frontal and somatosensory regions as well as between occipital and cingulate cortices. Changes in white matter integrity (as indexed by QA) were also in general agreement with observed pattern changes in the number of white matter fibers. Analysis of resting state sequences revealed patterns of increased connectivity between temporal and inferior frontal areas in the blind while decreases in functional connectivity were observed between occipital and frontal and somatosensory-motor areas and between temporal (mainly fusiform and parahippocampus) and parietal, frontal, and other temporal areas. Finally, correlations in white matter connectivity and functional connectivity observed between early blind and sighted controls showed an overall high degree of association. However, comparing the relative changes in white matter and functional connectivity between early blind and sighted controls did not show a significant correlation.

While extensive differences were observed along all three MRI-based modalities, the most notable finding was the evidence of increased inter- and intrahemispheric white matter connectivity in blind individuals. Taken together, the combination of these morphological, structural, and functional connectivity changes may underlie the neurophysiological substrate that supports crossmodal sensory processing and compensatory behaviors observed in individuals who are blind.

### Relationship to previous studies

Morphological changes in gray and white matter as well as subcortical structures have been investigated previously by a number of groups. In general, our differences in morphological findings from the exploratory analysis are in agreement with previous studies using both volume and surface based morphometry approaches investigating cortical and subcortical morphometry. We observed trends for co-occurring decreased cortical volume and increased cortical thickness within occipital cortex in the blind as compared to sighted controls as reported by other groups [34, 37–40, 80]. Specifically, we found increased occipital cortical thickness within the left lingual gyrus and bilateral lateral occipital regions [37, 39, 40], although these increases in cortical thickness did not survive correction for multiple comparisons in our sample. For areas that did survive correction for multiple comparisons, we found decreased cortical volume with pericalcarine calcarine cortex and decreased cortical thickness in fusiform cortex. A concomitant increase in volume with inferior parietal cortex was also noted. The observed decreased cortical and white matter volume within pericalcarine can be interpreted within the context of geniculo-calcarine transneuronal degeneration ([39]; see below for further discussion). However, the significance of morphological changes in higher order regions are less clear at this time. This is also of particular interest given the purported roles of these areas in the sighted namely; fusiform gyrus in face recognition [81] and inferior parietal cortex implicated in visual control of action [82].

Notably, we did not find statistically significant differences between both groups with regards to the morphometry of subcortical structures (including the hippocampus, amygdala, caudate, and putamen) although a previous group of studies did report increased hippocampal volume in the blind [35, 83, 84]. However, closer examination of these studies revealed that increases in volume were specific to the anterior (head) of the right hippocampus (with no changes in the body or tail) in one study [83] and decreases in the posterior right hippocampus in another study [85]. In our study, the hippocampus was segmented and measured as a single unit. Thus, it is possible that methodological differences may explain the observed discrepancy.

In contrast to previous reports, we found evidence of both significant increases and decreases in white matter connectivity (both inter- and intrahemispheric) in the blind compared to sighted controls. The vast majority of prior studies have highlighted evidence of marked reductions in the structural volume of numerous brain areas, and in particular, geniculocalcarine structures and tracts including the optic radiations [35, 36, 41, 42, 44]. Furthermore, decreases in overall FA within occipital cortical areas [42, 43, 86] have also been reported. These decreases in structural volume and integrity have been interpreted within the context of volumetric reductions observed within occipital cortical areas, possibly consistent with geniculo-calcarine transneuronal degeneration [39]. In contrast, the only reports of increases in white matter volume were observed in sensorimotor cortex [87] and evidence of increased white matter integrity within the cortical spinal tract [88]. Regional changes were also reported within the corpus collosum [35, 44]. Using a whole brain analysis approach, Shu and colleagues (2009) (employing DTI) reported an overall decrease in white matter connectivity in the blind including connections between occipital and inferior frontal areas [43, 86]. However, evidence of increased connectivity was observed within regions implicated with sensorimotor functioning [43]. In another study, Wang and colleagues (2013) compared changes in FA between congenital and late blind (i.e. after 18 years of age) individuals as well as in sighted controls [47], confirming findings of overall reduced white matter integrity within the optic radiations. However, increased FA was found within the corticospinal tracts in both blind groups as compared to sighted controls [47]. Finally, Reislev and colleagues (2016) used anatomical connectivity mapping based on DTI data obtained from congenitally blind subjects and found reduced anatomical connectivity in the splenium and mid-body of the corpus callosum, as well as decreased FA in the cortical spinal tract with no further evidence of increased anatomical connectivity [46].

As opposed to these aforementioned studies employing DTI, our study concentrated on white matter whole-brain connectivity patterns reconstructed by diffusion data acquired by HARDI. Using fiber number as the main outcome of interest, we found evidence of both increases and decreases in white matter connectivity in the blind compared to sighted controls. These observed white matter changes were also supported by analyzing an index of white matter structural integrity: quantitative anisotropy (QA). In particular, we found evidence of increased connections involving superior temporal cortex; a region of the brain that is involved in spatial awareness [89].

The reason for our ability to observe both significant increases and decreases in white matter connectivity (as opposed to only decreases as in previous studies) can likely be explained by the variant of the diffusion MRI approach used in this study. In contrast to previous studies carried out with blind individuals, we employed HARDI rather than DTI to characterize white matter connectivity and integrity. While both techniques provide information regarding the degree of water diffusivity in the brain in order to derive local axonal fiber orientation, DTI is unable to correctly resolve multiple individual orientations within the same voxel such as crossing fibers (note that it has estimated that one third of white matter voxels may be affected by this issue; see [90]). In contrast, HARDI collects diffusion weighted images with many more gradient directions (i.e. 64) compared to the minimum of six required for DTI. As such, it has become increasingly established that HARDI is superior to DTI in its ability to delineate crossing fibers and ultimately the overall microarchitecture of the brain [91–93].

Finally, with regards to resting state functional connectivity, our results are in general agreement with previous findings reporting patterns of both increases and decreases in connectivity in the blind. In a recent study, Burton and colleagues (2014) found evidence of decreased functional connectivity between occipital cortex and auditory as well as somatosensory cortices in early blind individuals as compared to normally sighted controls. In contrast, increased functional connectivity was reported between occipital and frontal cortical areas (specifically, dorsolateral prefrontal cortex) along with parietal regions of the brain [55]. Enhanced functional connectivity between occipital and frontal cortical areas implicated with executive control was reported, particularly with regards to working memory function (i.e. medial supplementary motor areas and prefrontal cortices, precentral sulcus, inferior frontal gyrus as well as superior frontal sulcus) [94]. Interestingly, we also noted increased functional connectivity between occipital structures and the frontal cortex (specifically, bilateral pars orbitalis in the inferior frontal gyrus) in our exploratory analysis. However, we observed decreased functional connectivity between occipital and somatosensory cortex and the medial orbitofrontal cortex after correction for multiple comparisons. Occipital connections to the medial orbitofrontal and the somatosensory cortices also showed decreased structural connectivity in our exploratory analysis, however these did not survive correction for multiple comparisons.

### Interpretation of findings within the context of neuroplasticity

As mentioned earlier, there has been considerable interest in relating compensatory behaviors in blind individuals within the context of observed structural and functional neuroplastic changes in the brain. While compensatory abilities in the blind have been reported across a wide variety of behavioral tasks and implicating different sensory modalities, it is important to note that these abilities are not universally evident across the blind population. Indeed, while there is evidence of enhanced sensory and cognitive task performance, there are also other reports suggesting that the blind are equal or even impaired on certain tasks compared to the their sighted counterparts [12, 13, 95]. This suggests that the absence of visual experience can induce either sensory compensation or the absence of calibration depending of the task-cognitive domain at play [96] (see also [53] for further discussion). Specifically, comparable (or even superior) perceptual and cognitive processing abilities in the blind (through the use of intact sensory modalities) would be in line with a “compensatory” hypothesis of neuroplasticity. If indeed adaptive behaviors observed in the blind are intimately related to changes in the overall structural and functional organization of the brain, evidence of increased morphological changes (e.g. gray matter volume or structural hypertrophy) and connectivity (white matter projections and functional connectivity) may be indicative of enhanced organization and facilitation of information processing occurring locally and/or between remote brain regions [97]. In light of this view, neuroplastic changes may be indicative of enhanced coordination between functional systems associated with heightened task performance. Our observation of enhanced intra- and interhemispheric white matter connectivity would be in line with evidence supporting the compensatory hypothesis. In contrast, impairment of perceptual performance and/or a lack of compensatory behaviors would be consistent with a “general loss” hypothesis. This in turn would be linked with potential decreases in brain morphometric and connectivity indices. In the same way, blind individuals with impaired crossmodal sensory and/or higher order cognitive functions may be explained within the context of associated decreases in morphometric and connectivity measures. Evidence of combined increases and decreases in functional connectivity throughout the brain (obtained from rsfcMRI) was also observed. This may be further suggestive of mixed compensatory and general loss mechanisms.

### Reconciliation of multimodal imaging results

In this study, we acquired spatially co-registered data from three MRI-based modalities; structural morphometry, HARDI, and resting state. When comparing across modalities, it is perhaps surprising to find evidence of inconsistencies between increased white matter connections and decreased functional connectivity in early blind and sighted individuals. In other words, while we observed increases in white matter connectivity in the blind, these connections were not always associated with concomitant enhancement in functional connectivity.

The reason for this apparent mismatch is not entirely clear at this time. However, such incongruities in observed structural and functional connectivity have been previously noted. A classic example is the evidence of strong interhemispheric functional connectivity between primary visual cortices despite a lack of direct white matter links between these same two regions [98]. While the interdependency between structural and functional connectivity is not yet well understood, it is also important to note that functional connectivity is largely a statistical concept (i.e. the temporal correlation of spatially remote signals as derived from fMRI based techniques; [99]). In contrast, anatomical connectivity describes physical pathways of information exchange between neural units. At the same time, anatomical connectivity derived from diffusion-based approaches does not speak to the temporal relationship between two structurally connected areas. Furthermore, the degree of functional connectivity is related to all the direct and indirect structural connections between the elements of a system. Thus, while strong functional connectivity between two areas may be suggestive of strong structural connectivity, it does not necessarily mean that the two areas are directly connected [100].

Recent work has also highlighted important differences in functional connectivity derived from resting state sequences with that of functional connectivity that characterizes task-specific activity. More specifically, it has been suggested in a number of reports that differences in connectivity patterns between populations are task dependent [101–103] and that task related and resting state functional connectivity in fact do not coincide. This provides further caution against inferring differences in functional integration between brain regions solely based on resting-state data. In this direction, it has been shown that whole-brain functional connectivity networks can fundamentally change in different task contexts (e.g. [104–106]), and thus are not constrained by networks identified solely by resting-state analyses. By extrapolation, white matter connectivity may perhaps be reflected by measures of effective connectivity (e.g. dynamic causal modeling; [107]). It has been demonstrated that changes in functional connectivity between occipital and temporal regions in blind individuals are indeed task dependent; while occipito-temporal connectivity at rest is typically lower in early blind [55], the connectivity between the exact same regions is higher in the blind while involved in a challenging auditory task [108].

To help resolve these inconsistencies, further studies should compare metrics of brain structure, white matter structural connectivity, along with measures of effective connectivity as well as individual parametric measures of behavioral performance. Again, by leveraging the advantages of multi-modal imaging, we are more likely to better understand how structure, connectivity, and behavior are reciprocally linked.

## Supporting information

S1 Fig Morphometry. Surface based morphometry analysis comparing differences in A) cortical volume and B) cortical thickness between early blind and sighted controls (corrected for multiple comparisons). Decreases in cortical volume (shown in blue) were evident within the left pericalcalcarine region, while increases (shown in red) were evident in right inferior parietal cortex. Decreases in cortical thickness were observed within left fusiform cortex (shown in blue). Differences in white matter volume and subcortical structures were not significant following correction for multiple comparisons. Abbreviations: L = left, R = right.
(EPS)

S2 Fig Analysis of Quantitative Anisotropy (QA) values. Quantitative anisotropy (QA) values revealed by HARDI. A) Exploratory analysis of ROI-pairs revealed an overall tendency for both increases and decreases in QA in ocular blind compared to sighted controls. Decreases were spread throughout both inter- and intra-hemispheric connections, whereas increases in QA were primarily limited to inter-hemispheric connections. B) Ball and stick representation of increases (upper panel) and decreases (lower panel) in QA values between early blind and sighted controls after FDR correction. Differences in connection strength are represented by line thickness, whereby thicker lines represent larger changes in the early blind group (based on p-value). Increases in QA in the early blind group are represented by orange lines, whereas decreases in QA in the ocular blind are represented by cyan lines. Dark blue spheres represent the nodes (i.e. ROIs) associated with the start and/or end points of the connections. Note the overall similarity to the pattern presented for white matter connectivity (fiber number) shown in Fig 2. Abbreviations: L = left, R = right, cing = cingulate, front = frontal cortex, par = parietal cortex, ins = insula, temp = temporal cortex, occ = occipital cortex.
(EPS)

S1 Table Surface Based Morphometry Analysis (uncorrected). (DOCX)

S2 Table HARDI QA (corrected). (DOCX)

## Abbreviations

HARDI: high angular resolution diffusion imaging
ROI: region of interest
rsfcMRI: resting state functional connectivity Magnetic Resonance Imaging
SBM: surface based morphometry
QA: quantitative anisotropy
