# Supplementary material for: Multimodal MR-imaging reveals large-scale structural and functional connectivity changes in profound early blindness
Source: PLoS One. 2017 Mar 22;12(3):e0173064. doi: 10.1371/journal.pone.0173064 (PMC5362049; doi:10.1371/journal.pone.0173064)
Supplement: S1 Table — (DOCX) [file pone.0173064.s003.docx]

| **Cortical Volume** | | | | | | | | | | | | | | | | |
| --- | --- | --- | --- | --- | --- | --- | --- | --- | --- | --- | --- | --- | --- | --- | --- | --- |
|  | Region label | uncorrected p value | Cluster Size (mm2) | MNI Coordinates | | | corrected p value |  |  | Region label | uncorrected p value | Cluster Size (mm2) | MNI Coordinates | | | corrected p value |
|  |  |  |  | x | y | z |  |  |  |  |  |  | x | y | z |  |
| **early blind < sighted controls** | | | | | | | | | | | | | | | | |
| **Left** | **pericalcarine**  caudal middle frontal superior parietal banks STS  middle temporal  rostral middle frontal pericalcarine  superior parietal precentral  rostral middle frontal rostral anterior cingulate rostral anterior cingulate precentral  lingual fusiform parahippocampal superior frontal superior frontal inferior parietal lingual  superior frontal precentral superior parietal  rostral anterior cingulate postcentral  pericalcarine | **0.0000**  0.0005  0.0000  0.0005  0.0008  0.0011  0.0012  0.0003  0.0010  0.0015  0.0016  0.0018  0.0018  0.0022  0.0028  0.0031  0.0031  0.0033  0.0035  0.0036  0.0039  0.0042  0.0043  0.0046  0.0046  0.0047 | **216.8200**  99.2100  77.0300  72.9300  48.6300  41.0700  25.4300  16.0400  11.3700  5.8900  17.7900  5.0800  9.0200  16.2700  12.7000  1.6500  7.9700  2.4300  3.6000  14.5200  1.6800  0.9300  5.2000  0.4700  0.5100  1.1900 | **-8.90**  -42.80  -7.70  -52.10  -46.50  -18.80  -12.90  -8.50  -57.30  -37.30  -6.50  -6.40  -52.00  -12.60  -42.60  -17.80  -16.70  -7.20  -50.10  -20.60  -7.00  -53.90  -21.30  -6.40  -53.70  -15.20 | **-91.00**  14.90  -67.80  -52.10  5.70  59.20  -80.00  -88.00  2.00  40.10  36.80  24.10  -5.40  -81.20  -57.70  -35.70  37.80  54.60  -60.20  -71.80  49.40  3.40  -63.10  38.60  -23.60  -75.80 | **7.10**  43.10  54.50  3.90  -34.70  -3.40  11.80  21.10  8.20  6.40  -2.30  -8.80  22.00  -6.60  -20.20  -9.50  46.60  25.70  34.70  -7.50  35.30  15.20  48.80  4.30  47.80  3.30 | **0.0066** n.s. n.s. n.s. n.s. n.s. n.s. n.s. n.s. n.s. n.s. n.s. n.s. n.s. n.s. n.s. n.s. n.s. n.s. n.s. n.s. n.s. n.s. n.s. n.s. n.s. |  | **Right** | pericalcarine superior parietal lingual  fusiform  lateral occipital inferior temporal superior temporal lateral occipital rostral middle frontal lateral orbitofrontal rostral middle frontal lateral orbitofrontal lateral occipital parahippocampal lingual  superior temporal superior parietal parahippocampal fusiform  lateral occipital lingual  superior temporal middle temporal | 0.0002  0.0007  0.0015  0.0006  0.0008  0.0014  0.0010  0.0012  0.0014  0.0016  0.0018  0.0019  0.0020  0.0022  0.0025  0.0032  0.0032  0.0038  0.0040  0.0045  0.0045  0.0046  0.0049 | 91.1500  57.1700  35.3800  34.9100  26.1000  26.5200  6.3700  9.6200  11.3700  16.2100  14.9300  5.9700  11.5400  2.0400  9.9800  3.8700  5.0100  0.4700  4.4300  2.9200  0.9300  1.8100  0.7000 | 14.80  9.50  17.40  36.70  44.50  51.90  44.40  29.70  23.10  26.40  33.20  26.20  40.40  22.90  26.20  52.60  34.00  23.20  35.70  40.00  33.40  64.80  54.70 | -78.20  -73.20  -67.40  -52.10  -75.70  -15.60  -32.50  -87.40  58.10  27.40  44.20  47.00  -81.00  -32.30  -61.30  -4.30  -46.00  -31.00  -44.10  -86.90  -49.70  -31.70  -9.90 | 4.80  49.30  2.70  -17.40  1.00  -29.70  -0.70  0.50  13.90  -13.80  20.00  -10.00  3.30  -11.30  0.80  -7.60  47.50  -12.60  -19.90  -11.90  -6.40  4.10  -26.30 | n.s. n.s. n.s. n.s. n.s. n.s. n.s. n.s. n.s. n.s. n.s. n.s. n.s. n.s. n.s. n.s. n.s. n.s. n.s. n.s. n.s. n.s. n.s. |
|  | | | | | | | | | | | | | | | | |
| **early blind > sighted controls** | | | | | | | | | | | | | | | | |
| **Left** | inferior temporal inferior temporal superior temporal  isthmus cingulate (retro spenial)  superior temporal entorhinal  lateral orbitofrontal precentral | 0.0013  0.0003  0.0009  0.0022  0.0039  0.0044  0.0048  0.0048 | 35.1900  25.7300  19.7700  3.5400  3.1700  0.5400  1.1100  1.1600 | -53.20  -48.10  -63.70  -7.90  -43.10  -25.30  -21.00  -24.40 | -37.70  -46.10  -41.60  -35.70  11.50  -8.40  36.50  -12.60 | -18.60  -15.80  12.00  28.90  -26.20  -31.40  -14.50  52.70 | n.s. n.s. n.s. n.s. n.s. n.s. n.s. n.s. |  | **Right** | **inferior parietal** inferior parietal insula  inferior parietal precentral superior frontal precuneus superior frontal superior parietal  medial orbitofrontal isthmus cingulate superior temporal medial orbitofrontal superior parietal inferior parietal  pars triangularis | **0.0001**  0.0007  0.0018  0.0007  0.0008  0.0009  0.0009  0.0018  0.0019  0.0020  0.0020  0.0022  0.0033  0.0033  0.0037  0.0047 | **241.2500**  35.4500  29.1900  21.2600  17.4500  13.8000  6.6800  3.7100  9.8500  6.8100  6.2300  3.3000  2.9500  4.9800  4.4000  0.6300 | **47.00**  42.10  36.90  44.70  33.20  10.00  7.90  6.60  17.50  7.00  5.30  57.70  8.30  33.40  39.20  49.60 | **-56.90**  -65.40  5.10  -56.40  -22.40  16.90  -52.40  45.00  -76.00  16.30  -33.70  3.00  15.20  -43.50  -58.40  31.30 | **14.20**  25.20  -11.60  24.80  57.70  42.50  60.80  42.00  42.90  -15.10  25.80  -8.40  -18.50  51.10  15.30  6.00 | **0.0032** n.s. n.s. n.s. n.s. n.s. n.s. n.s. n.s. n.s. n.s. n.s. n.s. n.s. n.s. n.s. |

| **Cortical Thickness** | | | | | | | | | | | | | | | | |
| --- | --- | --- | --- | --- | --- | --- | --- | --- | --- | --- | --- | --- | --- | --- | --- | --- |
|  | Region label | uncorrected p value | Cluster Size (mm2) | MNI Coordinates | | | corrected p value |  |  | Region label | uncorrected p value | Cluster Size (mm2) | MNI Coordinates | | | corrected p value |
|  |  |  |  | x | y | z |  |  |  |  |  |  | x | y | z |  |
| **early blind < sighted controls** | | | | | | | | | | | | | | | | |
| **Left** | **fusiform**  caudal middle frontal superior frontal  insula  superior parietal fusiform  pars opercularis  inferior parietal parahippocampal precentral parahippocampal rostral middle frontal supramarginal  caudal middle frontal lateral occipital middle temporal fusiform  fusiform  middle temporal lingual  insula  superior temporal inferior parietal | **0.0001**  0.0002  0.0002  0.0007  0.0012  0.0007  0.0008  0.0009  0.0011  0.0012  0.0018  0.0019  0.0024  0.0030  0.0030  0.0035  0.0039  0.0039  0.0041  0.0042  0.0047  0.0048  0.0049 | **143.7900**  98.5900  72.7900  53.6300  29.2500  13.8600  8.2100  8.5000  7.0900  21.9300  8.5400  9.9700  10.4700  4.6200  6.7000  5.1600  4.4400  8.9800  3.5200  2.9500  0.3600  0.4900  0.4000 | **-39.40**  -42.00  -19.10  -34.30  -8.60  -31.30  -35.50  -45.30  -17.80  -39.50  -18.50  -22.20  -58.10  -36.70  -41.80  -60.40  -31.80  -34.80  -59.30  -26.10  -35.20  -54.90  -41.60 | **-42.10**  13.10  32.50  -18.80  -68.10  -46.80  14.80  -64.00  -35.70  2.80  -40.20  48.20  -49.30  3.70  -74.70  -38.20  -53.20  -54.10  -13.50  -66.70  -5.20  -22.20  -65.30 | **-22.70**  43.00  44.00  3.20  56.00  -14.50  11.90  8.20  -9.50  12.80  -10.50  29.30  31.10  35.80  -4.20  -13.00  -7.90  -16.70  -20.00  2.90  11.60  0.40  12.90 | **0.0288** n.s. n.s. n.s. n.s. n.s. n.s. n.s. n.s. n.s. n.s. n.s. n.s. n.s. n.s. n.s. n.s. n.s. n.s. n.s. n.s. n.s. n.s. |  | **Right** | lingual  rostral middle frontal rostral middle frontal lateral occipital middle temporal superior frontal superior frontal  banks STS  lateral orbitofrontal pars orbitalis superior frontal inferior temporal  rostral middle frontal precuneus  inferior parietal lateral occipital banks STS fusiform  inferior parietal  precentral middle temporal superior temporal fusiform precentral  lingual  inferior temporal caudal middle frontal superior frontal supramarginal superior temporal pars triangularis supramarginal | 0.0004  0.0004  0.0008  0.0006  0.0009  0.0008  0.0008  0.0011  0.0012  0.0014  0.0014  0.0017  0.0017  0.0019  0.0020  0.0020  0.0026  0.0030  0.0031  0.0033  0.0034  0.0036  0.0036  0.0037  0.0037  0.0039  0.0043  0.0043  0.0043  0.0045  0.0047  0.0050 | 91.7100  68.6400  50.6200  35.3700  31.1200  16.6700  13.5900  11.4000  14.2800  5.3400  11.1600  17.7700  12.5100  9.4600  4.1900  15.9600  5.1500  5.6700  5.6000  2.7800  5.4000  2.2900  5.2000  1.4200  5.5500  5.3500  3.0800  2.2600  5.6100  0.9100  2.6500  0.4000 | 33.40  32.40  23.00  45.10  46.70  18.70  6.90  44.30  23.70  37.00  23.80  55.20  38.90  12.40  32.20  26.00  51.00  33.60  36.30  42.50  56.30  53.30  33.40  57.40  18.50  47.90  34.50  15.50  54.10  42.30  50.50  56.70 | -49.70  43.10  57.50  -75.90  8.20  38.60  6.50  -37.80  28.00  36.50  23.70  -17.00  51.50  -41.50  -64.80  -89.60  -35.10  -67.40  -84.30  3.90  -10.10  -35.60  -48.50  3.30  -77.50  -49.20  1.60  47.80  -22.40  -6.70  35.30  -21.60 | -6.40  20.40  14.40  1.30  -33.20  41.90  64.20  2.70  -11.80  -7.80  40.30  -33.90  -1.90  35.40  36.20  17.90  5.50  -14.70  15.10  11.60  -26.00  15.80  -17.40  6.90  -11.10  -14.40  48.80  31.40  21.80  -17.40  -1.50  22.30 | n.s. n.s. n.s. n.s. n.s. n.s. n.s. n.s. n.s. n.s. n.s. n.s. n.s. n.s. n.s. n.s. n.s. n.s. n.s. n.s. n.s. n.s. n.s. n.s. n.s. n.s. n.s. n.s. n.s. n.s. n.s. n.s. |
|  | | | | | | | | | | | | | | | | |
| **early blind > sighted controls** | | | | | | | | | | | | | | | | |
| **Left** | lingual entorhinal precuneus precentral lateral occipital precentral lingual paracentral  superior parietal | 0.0008  0.0002  0.0020  0.0022  0.0028  0.0028  0.0029  0.0047  0.0048 | 74.7900  26.0900  6.8700  4.1700  12.5200  5.2200  5.5300  0.4100  0.7900 | -13.80  -25.30  -6.10  -44.50  -10.60  -33.10  -4.40  -9.10  -32.50 | -66.60  -8.40  -59.90  -8.00  -99.00  -25.60  -74.20  -27.60  -45.00 | -2.50  -31.40  23.70  48.90  2.80  48.90  3.70  49.70  38.30 | n.s. n.s. n.s. n.s. n.s. n.s. n.s. n.s. n.s. |  | **Right** | precentral lateral occipital inferior temporal precuneus superior parietal lateral occipital superior parietal | 0.0001  0.0013  0.0014  0.0016  0.0038  0.0039  0.0047 | 38.5500  15.4000  10.2000  7.6000  2.3600  8.9800  0.7500 | 31.60  9.60  45.20  6.40  20.50  18.50  25.90 | -22.50  -98.30  -59.60  -44.80  -86.20  -93.80  -56.60 | 56.90  12.20  -8.60  43.00  27.30  -9.70  63.20 | n.s. n.s. n.s. n.s. n.s. n.s. n.s. |

**S1 Table.** Surface Based Morphometry Analysis (uncorrected)
