# Supplementary material for: Multimodal MR-imaging reveals large-scale structural and functional connectivity changes in profound early blindness
Source: PLoS One. 2017 Mar 22;12(3):e0173064. doi: 10.1371/journal.pone.0173064 (PMC5362049; doi:10.1371/journal.pone.0173064)
Supplement: S2 Table — (DOCX) [file pone.0173064.s004.docx]

| **QA (HARDI)** | | | | | | | | | | |
| --- | --- | --- | --- | --- | --- | --- | --- | --- | --- | --- |
| **early blind < sighted controls** | | | | | | | | | | |
| **Left Intrahemispheric** | | |  | **Interhemispheric** | | |  | **Right Intrahemispheric** | | |
| ROI 1 | ROI 2 | adjusted p value |  | ROI 1 | ROI 2 | adjusted p value |  | ROI 1 | ROI 2 | adjusted p value |
| **OCCIPITAL**  lh lateral occipital  **TEMPORAL** lh entorhinal lh banks sts  **FRONTAL**  lh pars triangularis | lh rostral anterior cingulate  lh insula  lh rostral middle frontal lh lateral orbitofrontal | 0.0065  0.0067  0.0201  0.0139 |  | **OCCIPITAL**  lh lateral occipital rh lingual  **PARIETAL**  lh superior parietal  **SENSORI-MOTOR**  lh postcentral rh postcentral rh postcentral | rh cuneus  lh rostral anterior cingulate rh precuneus  rh superior temporal  lh supra marginal  lh inferior temporal | 0.0152  0.0182  0.0104  0.0128  0.0237  0.0245 |  | **OCCIPITAL**  rh lingual  **TEMPORAL**  rh fusiform  **FRONTAL**  rh pars opercularis  **SENSORI-MOTOR**  rh precentral | rh parahippocampal rh retrosplenial  rh posterior cingulate  rh entorhinal | 0.0129  0.0229  0.0244  0.0002 |
|  | | | | | | | | | | |
| **early blind > sighted controls** | | | | | | | | | | |
| **Left Intrahemispheric** | | |  | **Interhemispheric** | | |  | **Right Intrahemispheric** | | |
| ROI 1 | ROI 2 | adjusted p value |  | ROI 1 | ROI 2 | adjusted p value |  | ROI 1 | ROI 2 | adjusted p value |
| **TEMPORAL**  lh superior temporal lh fusiform  lh fusiform | lh rostral middle frontal lh lateral orbitofrontal lh inferior temporal | 0.0050  0.0101  0.0129 |  | **TEMPORAL**  rh superior temporal | lh retrosplenial | 0.0102 |  | **FRONTAL**  rh pars orbitalis | rh insula | 0.0228 |

**S2 Table.** HARDI QA (corrected)
